# Front-Face Fluorimeter for the Determination of Cutting Time of Cheese Curd

**DOI:** 10.3390/foods10030576

**Published:** 2021-03-10

**Authors:** Maryna Lazouskaya, Irina Stulova, Aavo Sõrmus, Ott Scheler, Kalle Tiisma, Toomas Vinter, Roman Loov, Martti Tamm

**Affiliations:** 1 Center of Food and Fermentation Technologies (CFFT), Akadeemia tee 15a, 12618 Tallinn, Estonia; irina.stulova@tftak.eu (I.S.); aavo@tftak.eu (A.S.); martti@tftak.eu (M.T.); 2 Department of Chemistry and Biotechnology, Tallinn University of Technology, Ehitajate tee 5, 19086 Tallinn, Estonia; ott.scheler@taltech.ee; 3 Tehnolabor OÜ, Tähetorni tn 21, 11625 Tallinn, Estonia; kalle@tehnolabor.ee (K.T.); toomas@tehnolabor.ee (T.V.); 4 Senmark Invest OÜ, Peterburi tee 46, 11415 Tallinn, Estonia; roman.loov@joeston.ee

**Keywords:** milk coagulation, front-face fluorescence, rheology, Berridge testing, *Rhizomusor Myhea*

## Abstract

The yield of product (cheese) during the cheese-making process depends on the cutting time of the cheese curd. However, the determination of optimal cutting time on an industrial scale is difficult as current standard methods are destructive or analyse only small volumes and not the entire milk to be curdled into cheese. This paper presents a novel front-face fluorimeter (FFF) that is designed to be immersed into a milk batch to enable the determination of the cutting time of cheese curd without the destruction of the sample. The FFF sensor signal corresponds to physical changes in milk during cheese formation and has high predictive power (*r* > 0.85) and good accuracy (RSE = 30%, considering daily variation between milk samples). The performance of the presented fluorimeter was on par with standard rheological and Berridge methods.

## 1. Introduction

The utilisation of milk for cheese production has been known since ancient times. Modern cheese production includes over 2000 types of cheese. Cheese is produced by coagulation of milk: a colloidal system consisting of casein in water. The system is thermodynamically unstable and tends to collapse when κ-casein is destabilised. This is followed by the aggregation of the micelles into clusters and the formation of gel (cheese curd) [1].

Currently, four methods of milk coagulation are known and used in cheese production: acid coagulation, acid and heat coagulation, rennet coagulation and coagulation with calcium chloride. At present, rennet is used to produce over 30% of cheese globally and its market is predicted to grow [2]. Rennet coagulation is defined as the clotting of milk caused by the addition of rennet enzymes. The mechanism of rennet coagulation has been described as a two-phase process [3,4]: (1) the primary enzymatic phase—hydrolysis of κ-casein accompanied by the release of caseinomacropeptide (CMP), and (2) the secondary coagulation phase—formation of gel and aggregation of destabilised micelles modified by rennet. The source of rennet is the stomachs of young calves, lambs or goatlings up to 10 days old. Production of rennet is expensive and inadequate for modern cheese manufacturing; moreover, it has several drawbacks. An alternative to rennet is non-animal enzymes, for example, microbial enzymes that are mainly products of fungal fermentation. Among them, *Rhizomucor Myhea* (also *Mucor miehei*) is the most studied [5].

Previously, it was reported that cheese yield is connected to curd cutting time, among other parameters [6]. Cutting the curd when the gel is too soft will lead to low cheese yield. However, cutting the curd when it is already firm will lead to cheese of poor quality with high moisture content.

Methods used in laboratory research to monitor milk coagulation and to determine the time to cut the curd can be divided into two groups: destructive and non-destructive [7]. The first group of methods is represented by optical methods (e.g., the Berridge method [8]), traditional methods (knife and finger tests) and rheological measurements [3]. The second group of methods is more diverse and is represented by the electrical method (measurement of conductivity [9]), thermal method (hot wire method [10]), ultrasonic method (pulse reflection technique [11]), optical methods (fluorescence spectroscopy [12,13], Fourier-transformed infrared spectroscopy [14,15], near-infrared light backscatter [16]), etc. [7]. However, all the above-mentioned methods are not compatible with industrial on-line and in-line application.

At the industrial level, the cutting time of cheese curd is determined by specially trained personnel, thus creating variability between cheese products. Nowadays cheesemakers wait for up to 30 min after adding the enzymes to milk to cut the curd. However, the factors that influence cheese firmness vary depending on the milk source and treatments [3]. Thus, cutting the coagulum 30 min after enzyme addition may not always be beneficial. Methods currently used in industry for optimum cutting time determination have out-of-control variations in process conditions [7].

Novel solutions in the industry that are designed to substitute specially trained personnel are based on (i) near-infrared devices (Coagusens^TM^, Chr. Hansen A/S, Denmark), (ii) light backscatter sensors (coagulation sensor for Tetra Pak^®^ Cheese Vats, Tetra Pak^®^, Sweden and CoAguLite, Reflectronics Inc., Lexington, KY, USA), and (iii) a sensor that measures infrared and fluorescence signals simultaneously (FluorLite-MC, Reflectronics Inc, USA). However, Coagusens^TM^ is designed for at-line application (analysis of a small portion of milk that is not necessarily a good representative for the whole milk batch), and Tetra Pak^®^ and Reflectronics Inc. sensors are designed to be mounted in the wall of the cheese vat [17], which limits the application of the sensors to the sizes and volumes of cheese vats, the number of sensors that can be used and the sensors’ mobility.

Therefore, there is a need for user-friendly portable solutions for monitoring milk coagulation in vessels of different size and shape, making them suitable for both small factories and large plants. In this work, we propose a sensor based on front-face fluorescence measurement as a possible solution for this purpose. The sensor allows monitoring of tryptophan fluorescence response as an indicator of the changes in milk during renneting. Fluorescence spectra of milk are rather difficult to interpret because of the complex composition of milk. The main fluorophores in milk can be divided into three groups: fluorophores in proteins, fluorophores in fat and fluorophores in vitamins [18]. However, during the renneting of milk, the principal physical changes involve only casein as the dominant (80–83%) protein of milk. The casein fluorescence response is determined by the presence of two aromatic amino acids: tryptophan and tyrosine. However, for tyrosine in casein, fluorescence intensity quenching has been observed [18,19].

Fluorescence techniques are known to be sensitive, rapid, and non-invasive analytical methods that provide information about samples on a molecular level. However, conventional and synchronous fluorescence measurements are not suitable for measurement in dairy products; their application is limited to diluted and transparent samples [20,21]. Compared to classical and synchronous fluorescence measurements, front-face fluorescence measurement can be applied to measurement in turbid samples [22], such as dairy products, since the angle formed between the excitation and emission beams is 90 degrees, which minimises the reflection of light, scattered radiation and depolarisation phenomena.

In the present work, we demonstrate our portable front-face fluorimeter (FFF) and compute a model for the determination of cheese cutting time based on its measurements.

## 2. Materials and Methods

### 2.1. Fabrication of Front-Face Fluorimeter Prototype

**Figure 1:**
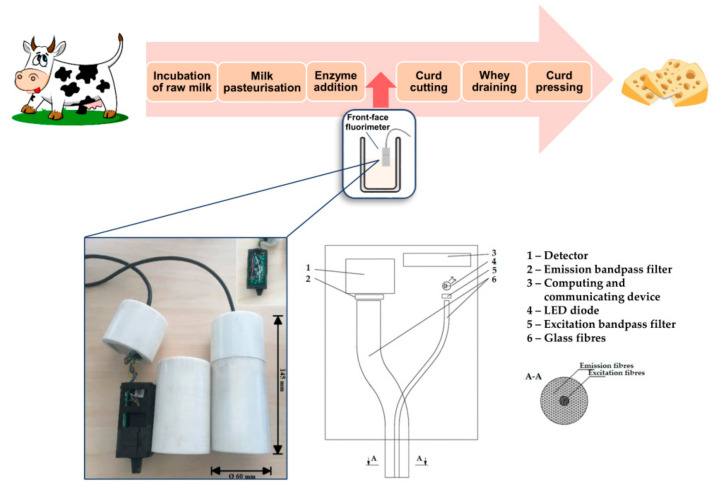
Schematic of the front-face fluorimeter (FFF) prototype application in the cheese-making process. Our prototype consists of a measuring device (schematic representation, bottom right) placed into a polycarbonate container (bottom left). The FFF prototype was vertically submerged in milk in such a way that approximately 75% of its length was covered with milk. However, the positioning of the FFF prototype did not influence the measurement. The drawings of a cow and cheese were taken from free license stock http://imgpng.ru/, accessed on 9 March 2021.

The FFF prototype was made with the following characteristics (Figure 1):

- a diode with an optical bandpass filter of λ = 285 nm in a 20 nm window (60 dB/dec) for excitation
- a photomultiplier with an optical bandpass filter of λ = 337 nm in a 10 nm window (60 dB/dec) for emission detection
- 700 Hz sinusoidal modulation of excitation and emission to filter the signal from ambient radiation
- glass fibres as a signal carrier.

All the equipment was placed in a polycarbonate container to meet the sanitary requirements of a dairy factory. To calibrate the FFF prototype, aqueous solutions of tryptophan in the dilution series of 3.3 × 10^−5^, 5 × 10^−5^, 5 × 10^−4^, 2 × 10^−4^, and 1 × 10^−4^ mg/mL were used.

### 2.2. Milk Samples

#### 2.2.1. Milk Samples for Preliminary Sensitivity Evaluation

Before measuring real-life samples, the prototype was tested on commercially available milk samples, varying in fat content and homogenisation state (Table 1). Homogenisation of milk leads to changes in fat globule size and therefore affects the physical properties of milk [23]. All milk samples were placed in a thermostatic water bath (Julabo^TM^, Thermo Fisher Scientific, Waltham, MA, USA) at 32 ± 0.2 °C prior to any measurements.

#### 2.2.2. Reconstructed Skim Milk

Reconstructed skim milk (RSM) was prepared as follows: first, 10 g of skimmed milk powder (Valio Ltd., Turku, Finland) was dissolved in 100 mL of Milli-Q water to yield a final concentration of 10% (*w/v)*. An aqueous solution of CaCl_2_ was added to the solution of skimmed milk to produce a final concentration of 5 mM. The resulting mixture was thoroughly stirred for 1 h at room temperature.

#### 2.2.3. Industrial Milk Samples

Industrial milk samples were received from the Saaremaa dairy factory (Saaremaa Piimatööstus AS, Kuressaare, Saare maakond, Estonia) at different times of the year. Sample characteristics derived from the dairy factory laboratory are presented in (Table 2). The fat and protein contents of milk samples were determined by infrared analysis (Mira Infrared Milk Analyzer, Bruker BioSpin, Billerica, MA, USA). All the milk samples were pasteurised at the factory at 72–75 °C for 20 s. Each milk sample was utilised for 9 measurements in total. Prior to utilisation, milk samples were stored at 4 °C.

### 2.3. Enzyme

One gram of Marzyme^TM^ (coagulating activity (IDF 157 A/1997)–2080–2305 IMCU gram^−1^, *Mucor miehei* protease concentration ≥28,000 mg/kg, Danisco, Épernon, France) was dissolved in 100 mL of distilled water and stored at 4 °C prior to utilisation. For all the experiments, the enzyme was added at a ratio of 100 μL of enzyme aqueous solution per 10 mL of milk. To study the influence of the enzyme concentration on the sensor response, aqueous solutions with concentrations in the range of 0.50, 0.75, 1.00, 1.25, and 1.50 g/L were used.

### 2.4. Berridge Testing

The Berridge test [8] was used for the preliminary determination of the clotting time of milk. Briefly, two glass tubes containing 10 mL of thermostated milk (32 ± 0.2 °C) each were used for the parallel determination of the clotting time. After 100 μL of the enzyme was added to each tube, a change in the aggregation state was observed and compared with the reference tube containing milk without enzyme. The clotting time (t_B_) was determined as the point of flocculation. The measurement was repeated three times.

### 2.5. Dynamic Rheology

The physical changes in milk were monitored with low-amplitude oscillatory rheometry using a Physica MCR 301 rheometer (Anton Paar GmbH, Graz, Austria). Furthermore, the direct strain oscillation option, the Peltier temperature control unit (C-PTD200) and the coaxial cylinder measuring system CC27 (outer diameter: 28.92 mm; inner diameter: 26.66 mm) were utilised.

For measuring purposes, 200 µL of the enzyme was added to 20 mL of thermostated milk, stirred and transferred into the measuring system. The coagulation temperature was 32 °C, and rheological parameters were monitored at 10-s intervals for 45 min. Rheological parameters were determined in oscillation mode at a frequency of 1 Hz and a strain of 0.01 (linear viscoelastic region, no perturbation of the coagulation process). During the measurement the elastic modulus (G’), viscous modulus (G”) and loss tangent (tan δ) were recorded. The gelation time (t_g_) was defined as the intersect between G’ and G” (or time of tan δ = 1). The cheese cutting time (t_cut_) was determined as the point at which the viscous modulus reached 20 Pa [24].

### 2.6. Fluorescence Measurement

#### 2.6.1. Fluorescence Measurement of Tryptophan

Preliminary validation of the FFF prototype was conducted on tryptophan aqueous solutions. To validate the FFF prototype performance as a fluorescence measuring device, the results derived from the prototype were compared with the results derived from the front-face fluorescence spectrometer Instant Screener (LDI, Tallinn, Estonia) and multipurpose spectral analyser Scalar Fluo Imager M53 (Scalar Analytical B.V., Breda, The Netherlands).

#### 2.6.2. Fluorescence Measurement of Milk Samples

For measuring purposes, 10 mL of the enzyme was added to 1 L of thermostated milk. After careful stirring, two FFF prototypes were placed in milk for parallel measurements. Milk samples were kept at 32 ± 0.2 °C in a water bath (JulaboTM, Thermo Fisher Scientific, Waltham, MA, USA) until measurement termination after monitoring for 50 min. Fluorescence intensity data were gathered at 1-s intervals. Excitation of tryptophan was carried out at 285 nm, and the emitted fluorescence signal was measured at 337 nm. The fluorescence signal was registered and monitored with the help of the specially designed program “SFS online” (Figure S1). The program utilises a custom code that is available upon request. The code is based on an algorithm developed in MATLAB (version R2016b, The MathWorks, Inc., Natick, MA, USA). The algorithm was designed to smooth the raw tryptophan fluorescence intensity data and to derive coagulation time parameters from it. The Savitzky–Golay smoothing filter [25] (polynomial order: 2; frame length: 150) was used to reduce the amount of the high-frequency noise component in the signal. Another reason to use the Savitzky–Golay filter was to substantiate the accuracy of the estimated parameters: the Savitszky–Golay filter retains features of the original signal (relative maxima, minima and width). The filtering was followed by the calculation of the filtered signal derivatives and the determination of the minimum of the second derivative of the tryptophan fluorescence signal. The time corresponding to the minimum of the second derivative of the tryptophan fluorescence profile (t_2min_) was used as a coagulation time parameter. Employment of the second derivative of the fluorescence signal helps to eliminate the decline in the signal caused by the presence of fat in milk. Furthermore, the application of the second derivative of the fluorescence signal was previously reported to gather more detailed information about changes occurring in milk during renneting [26]. The calculated t_2min_ were compared with the time parameters determined by the Berridge and rheological methods described above.

### 2.7. Statistical Analysis

For coagulation measurements, the fluorescence intensity of milk was recorded by two FFF prototypes in parallel. Analysis of variance (ANOVA) and t-test were applied to estimate the significance of (i) the difference between two prototype readings and (ii) the difference in consequent sample readings. To evaluate the correlation between all the acquired data, regression analysis was conducted on the following datasets: t_2min_–t_B_, t_2min_–t_g_ and t_2min_–t_cut_. Correlations between the variables were evaluated using the Pearson correlation coefficient. Furthermore, the residual standard error (RSE) and root mean square error (RMSE) were used as relative and absolute indicators, respectively, to evaluate how well the model fits the data.

The fluorescence intensity of milk was recorded by two FFF prototypes in parallel. To estimate if the readings from the prototypes could be united into one dataset, the difference between the two prototype readings was analysed with ANOVA and t-test. For the analysed data, the p-value was higher than the 0.05 significance level, indicating that the variances of the datasets for the sensors were the same and allowing us to combine the data from the two prototypes into a joint dataset. Thus, each point on the graphs discussed in Section 3.3 corresponds to the mean value of 6 measurements for t_2min_ and t_B_ and the mean value of 3 measurements of t_g_ and t_cut_.

## 3. Results

### 3.1. Validation of the FFF against Traditional Fluorimeters

During the renneting, changes in micelle structure and protein interaction take place; caseinomacropeptide is released from casein micelles as a result of κ-casein hydrolysis [3,13]. When a sufficient amount of κ-casein is hydrolised, the revealed *para*-κ-casein micelle surface starts to aggregate and eventually forms a solid-like viscoelastic gel network [3]. These changes in micelle structure and protein interaction cause changes in the tryptophan environment, which can be monitored with fluorescence measurement [27,28,29] since the fluorescent properties of tryptophan in hydrophobic and hydrophilic environments are different [19,20]. First, the FFF prototypes were validated against traditional benchtop fluorimeters by conducting measurements in standard aqueous solutions of tryptophan. Excitation of tryptophan was carried out at 285 nm, and measurement of the emitted fluorescence signal was measured at 337 nm. The measurements showed a good correlation with commercially available systems (*r* > 0.99) within the investigated concentration range.

### 3.2. Preliminary Sensitivity Evaluation

**Figure 2:**
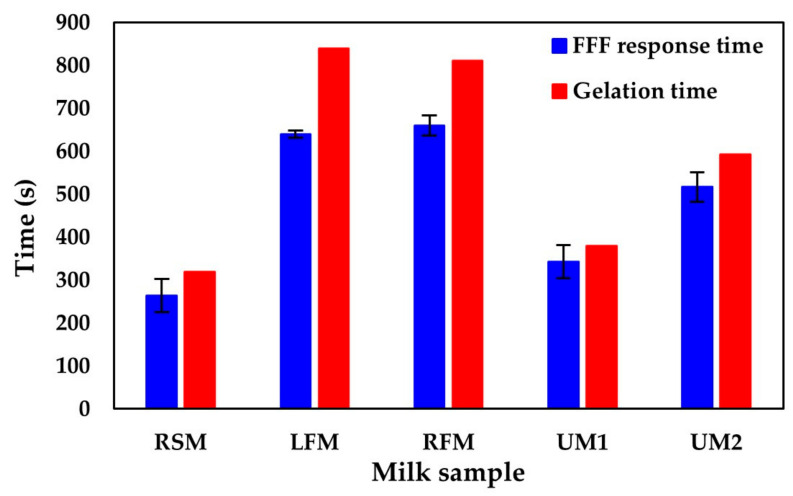
The FFF prototype is suitable to measure coagulation in different milk samples. Time-dependent parameters (Y-axis) were evaluated for milk samples with different fat content (X-axis). The results obtained from the FFF prototype (minimum of the second derivative of the fluorescence signal; blue bar) were not different from the rheological results (gelation time; red bar) for RSM, UM1 and UM2 and were only slightly different for LFM and RFM (the percentage difference was less than 4%). Error bars represent the standard deviation of the mean of 3 measurements.

Second, the FFF prototypes were tested on milk samples of different fat content and homogenised state. For a better understanding of the changes in the tryptophan fluorescence signal during rennet coagulation, dynamic rheological measurements and fluorescence profile registration were conducted simultaneously. The results of the measurements are presented in (Figure 2). The fluorescence and rheological profiles can be found in the Supplementary Material, (Figure S2). For both the RSM and unhomogenised milk samples, UM1 and UM2, the t_2min_ points overlapped with the t_g_ points; the results from homogenised milk samples, LFM and RFM, showed only a slight difference in values.

### 3.3. Fluorescence Measurement of Industrial Milk Samples

**Figure 3:**
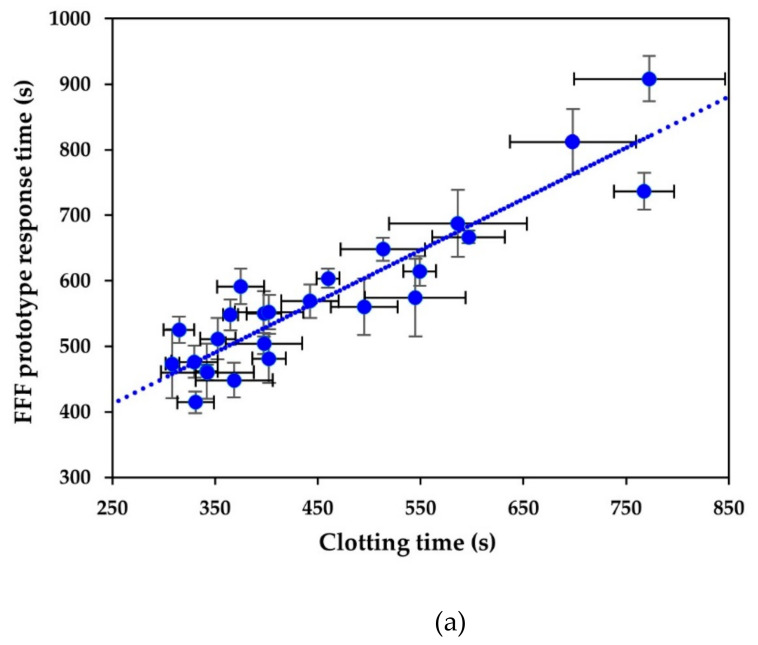

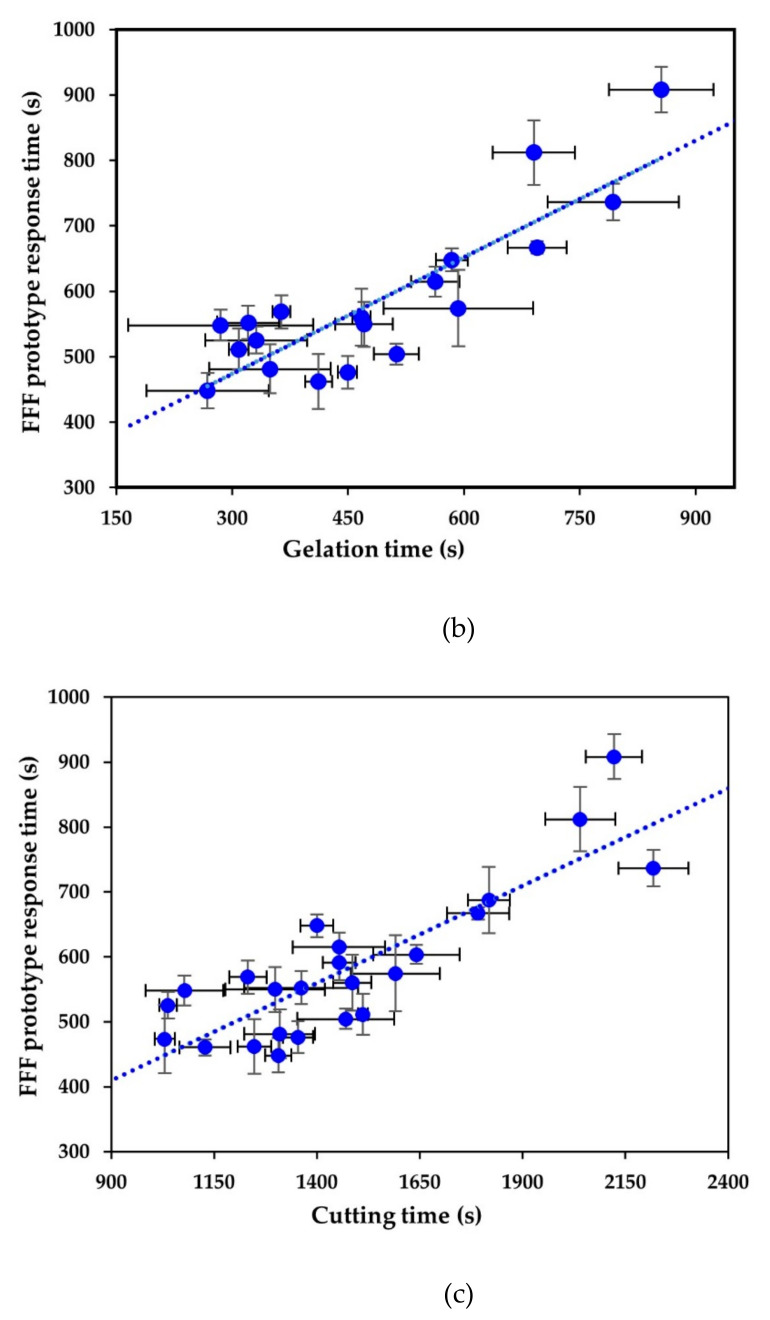
The FFF prototype detects physical changes in milk during renneting. The FFF prototype signal (t_2min_) (Y-axis) is strongly correlated with physical parameters detected by conventional methods (X-axes): (**a**) clotting time (t_B_), (**b**) gelation time (t_g_) and (**c**) cutting time (t_cut_). Error bars represent the standard deviation of the mean of 3–6 measurements. Linear correlations (*r* > 0.8) were observed in all the cases with adequate error not exceeding 63 s for samples varying in fat content.

Third, we conducted measurements of industrial milk samples. To evaluate the correlation between the acquired data, regression analysis was conducted on the following datasets: t_2min_–t_B_, t_2min_–t_g_ and t_2min_–t_cut_ (Figure 3).

In all cases, there was a strong positive correlation (*r* > 0.8) (Table 3). Clearly, t_2min_ was different from clotting time (t_B_) and highly correlated. The linear correlation of t_2min_ and t_B_ resulted in a root mean standard deviation of prediction of 48 s, which can be viewed as acceptable as the relative standard error is 16%. However, in the case of the correlation analysis between t_2min_ and rheological results (t_g_ and t_cut_), RSE was higher than 25% (Table 3), which might indicate high sampling error. This can be explained by the fact that unhomogenised milk is used for cheese production. Thus, fat particles differ in size and disperse unevenly throughout the milk, causing the variation in coagulation speed. Additionally, the measurements were performed on samples varying in fat content (protein-to-fat ratio varied from 0.92 to 1.38, Table 4). The obtained equations take into consideration the difference in daily variation between samples and could be applied to all the samples corresponding to the abovementioned range of protein-to-fat ratio.

The final aim of the implementation of the sensors was to determine the cutting time from the fluorescence measurement. For this purpose, t_cut_ was expressed as a function of t_2min_:

t_cut_ = 1.24·t_2min_ + 238.29 (s) (1)

### 3.4. Coagulant Concentration

**Figure 4:**
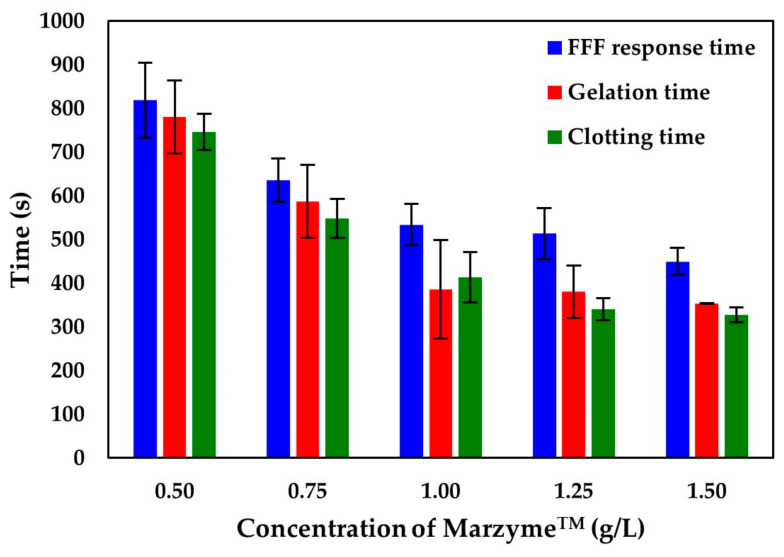
The FFF prototype shows that the optimum coagulant concentration is 1 g/L. All the time-dependent physical characteristics (Y-axis) decreased with increasing Marzyme^TM^ concentration (X-axis): blue bar—FFF prototype signal (t_2min_); red bar—gelation time (t_g_); green bar—clotting time (t_B_). The coagulation time of 1 g/L remained approximately the same. Error bars represent the standard deviation of the mean of 18–60 measurements.

Enzymes are indisputably important in cheesemaking. Enzymes influence the cheese produced in two ways: (i) by hydrolysis of κ-casein (affects clotting) and (ii) because of its proteolytic activity (affects the ripening of cheese). In our work, we tested the optimum concentration for coagulant Marzyme^TM^ in a range from 0.50 to 1.50 g/L. The dependence of coagulation time on the concentration of the enzyme is presented in (Figure 4). At low enzyme concentrations, the coagulation time was the longest and did not significantly change at concentrations of the enzyme above 1 g/L (Figure 4). This pattern was noticed for all the determined time constants: t_2min_, t_B_, t_cut_, and t_g_. Therefore, the concentration of 1 g/L is sufficient to reach faster coagulation of milk from the point of physical parameters.

## 4. Discussion

### 4.1. The FFF Prototype Showed a Strong Positive Correlation with Traditional Fluorimeters

The main feature that differentiates FFF prototypes from traditional fluorimeters is that FFF prototypes are able to conduct measurements only at specific excitation and emission wavelengths. However, as tryptophan has only one peak in fluorescence spectra [19], for tryptophan detection, a single wavelength measurement is sufficient. This fact along with the high correlation of the FFF prototypes performance with that of traditional benchtop fluorimeters (*r* > 0.99), allows us to conclude that our FFF prototypes can be used for fluorescence measurement of tryptophan in milk samples.

### 4.2. Front-Face Fluorimeter Prototype Is on Par with Rheology for Coagulation Studies in Milk with Different Fat Contents

The general pattern of the tryptophan fluorescence evolution during the renneting of modelled milk (see the Supplementary Material) was similar to that previously reported [12].

According to McMahon et al. [30], there is a lag time that is associated with the initial formation of aggregated nuclei material and consequent aggregation of the nuclei. Therefore, clotting time is determined as the point of network formation. In the case of Berridge testing, visual clotting time is observed (clotting time is determined as the visual appearance of flocks), while in the case of rheological or fluorescence measurements, actual clotting time is observed (clotting time is determined as the change in physical parameters registered by the device).

### 4.3. The FFF Prototype Is Able to Detect Physical Changes in Industrial Milk Samples during Renneting

The milk used for cheese making in the industry varies in several parameters: fat and protein content, pH, lactose, non-fat solids content, etc. Among them, fat and protein content were shown to have an influence on cheese cutting time [9,31,32,33], composition and yield [34]. Variability of milk samples can be reduced by standardisation of the protein-to-fat ratio [35].

Previously, Calvo [32] showed that cutting time was higher for samples of whole milk than for skimmed milk. The author suggested that fat content prevents the enzyme action of the first step of micelle aggregation [32]. This corresponds well with our results. However, for t_g_ an inverted relationship was observed (Table 4). Apparently, t_g_ is more sensitive to the first step of coagulation, that is, the hydrolysis of κ-casein. Felfoul et al. [9] observed the same tendency when studying whole and low-fat milk. The authors suggested that this difference can be attributed to the membrane nature of fatty globules or emulsion size. Emulsion size might modify the possible interactions between κ-casein and milk fat, therefore influencing the accessibility of κ-casein to the enzyme [9].

## 5. Conclusions

In this study, a front-face fluorimeter prototype was evaluated for application as a sensor for monitoring rennet-induced coagulation of milk. The proposed device was designed to be used as an indicative instrumental measurement as an alternative to the standard cheesemaker knife test. A model for predicting cheese cutting time was developed based on time parameters extracted from the prototype. The limitations of the application of the discussed device are connected to the cleaning of the prototype after each measurement (the prototype needs a thorough cleaning, especially around the detector) and troubleshooting in the microprocessor (to extract the microprocessor, the container needs to be cut open and then substituted for a new one). Future plans include the implementation of the prototype for in-line monitoring of the rennet coagulation process at the dairy factory.

## Figures and Tables

**Table 1:** Characteristics of milk samples used for preliminary sensitivity evaluation.

Sample	Pasteurisation	Name	Fat Content %
Reconstituted Skim Milk (Valio Ltd., Finland)	Low temperature	RMS	0
Homogenised Low Fat Milk (Valio Ltd., Finland)	Ultra-high temperature	LFM	<0.05
Homogenised Regular Fat Milk (Valio Ltd., Finland)	Ultra-high temperature	RFM	2.5
Unhomogenised Milk (Nopri dairy farm, Estonia)	Raw milk	UM1	1.6
Unhomogenised Cheese Milk (Saaremaa dairy factory, Estonia)	Low temperature	UM2	2.5

**Table 2:** Characteristics of industrial milk samples (Saaremaa Piimatööstus AS, Estonia).

Sample No.	Fat Content %	Protein Content %	Protein-to-Fat Ratio
1	2.80	3.30	1.18
2	2.40	3.30	1.38
3	2.40	3.30	1.38
4	2.80	3.30	1.18
5	2.40	3.30	1.38
6	2.40	3.30	1.38
7	3.62	3.30	0.91
8	2.90	3.42	1.18
9	2.90	3.30	1.14
10	2.90	3.49	1.20

**Table 3:** Values of the correlation coefficient, coefficient of determination, residual standard error (RSE) and root mean square error (RMSE) for the relation between t_B_, t_g_, t_cut_ and t_2min_.

	t_2min_–t_B_	t_2min_–t_g_	t_2min_–t_cut_
Equation	t_2min_ = 0.78·t_B_ + 216.55	t_2min_ = 0.61·t_g_ + 286.15	t_2min_ = 0.29·t_cut_ + 150.13
Coefficient of determination (*r^2^*)	0.85	0.75	0.72
Correlation coefficient (*r*)	0.92	0.87	0.85
RMSE, s	48	58	63
RSE, %	16	26	30

**Table 4:** Mean values of t_g_, t_B_, t_2min_ and t_cut_ for milk samples with different protein-to-fat ratio (enzyme concentration—1 g/L).

Samples No.	Protein-to-Fat Ratio	t_g_, s	t_B_, s	t_2min_, s	t_cut_, s
7	0.92	183	460	604	1642
1–6	1.14–1.18	329	403	538	1336
8–10	1.38	349	373	503	1302

## Data Availability

Data is contained within the article or the Supplementary Materials. The code and the software of “SFS Online” is available upon request from the authors.

## Supplementary Materials

The following is available online at https://www.mdpi.com/2304-8158/10/3/576/s1, Figure S1: Interface of the program ‘SFS online’, Figure S2. Rheological and front-face fluorescence profiles for: (a) RSM; (b) LFM; (c) RFM; (d) UM1; (e) UM2. Tryptophan fluorescence data (black dash line) recorded during coagulation of milk and the corresponding second derivatives (black line), the evolution of elastic modulus G’ (blue line) and viscous modulus G” (red line) during rennet coagulation. The black vertical line indicates the minimum of the second derivative and the red vertical line indicated the intersection of elastic and viscous moduli (gelation time). In the case of UM2 no intersection of elastic and viscous moduli was observed; the green vertical line indicates apparent gelation time.
