# Supplementary material for: Front-Face Fluorimeter for the Determination of Cutting Time of Cheese Curd
Source: Foods. 2021 Mar 10;10(3):576. doi: 10.3390/foods10030576 (PMC7999451; doi:10.3390/foods10030576)
Supplement: Supplementary file 1 [file foods-10-00576-s001.pdf]

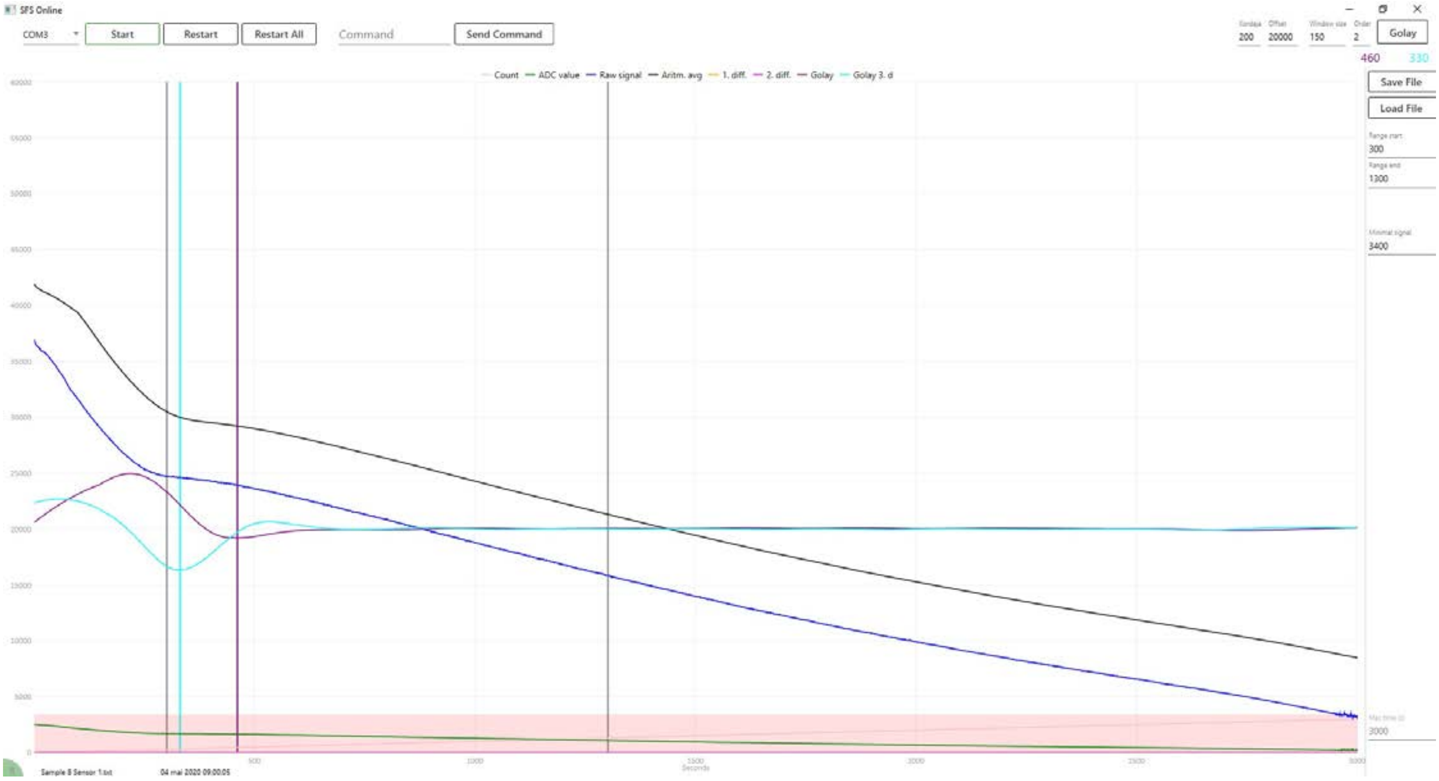

Figure S1. Interface of the program "SFS online".

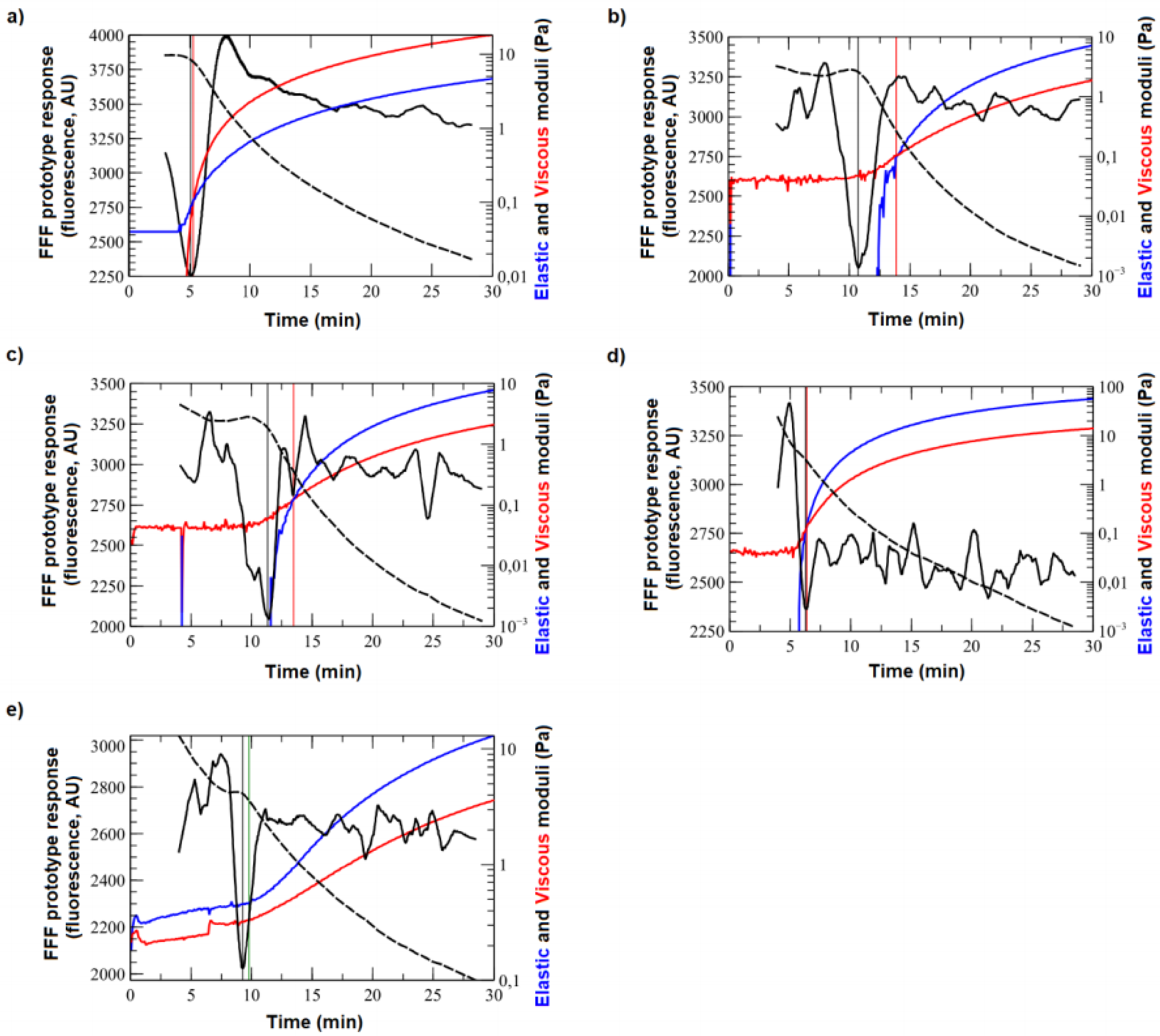

Figure S2. Rheological and front-face fluorescence profiles for: (**a**) RSM; (**b**) LFM; (**c**) RFM; (**d**) UM1; (**e**) UM2. Tryptophan fluorescence data (black dashed line) recorded during coagulation of milk and the corresponding second derivative (black line), the evolution of elastic modulus G′ (blue line) and viscous modulus G″ (red line) during rennet coagulation. The black vertical line indicates the minimum of the second derivative and the red vertical line indicated the intersection of elastic and viscous moduli (gelation time). In the case of UM2, no intersection of elastic and viscous moduli was observed; the green vertical line indicates apparent gelation time.
